# Ten promotion and tenure tips for microbiologists and immunologists

**DOI:** 10.1128/mbio.00631-24

**Published:** 2024-03-29

**Authors:** Jacob S. Yount

**Affiliations:** 1 Department of Microbial Infection and Immunity, Viruses and Emerging Pathogens Program of the Infectious Diseases Institute, The Ohio State University, Columbus, Ohio, USA; Johns Hopkins Bloomberg School of Public Health, Baltimore, Maryland, USA

**Keywords:** microbiology, immunology, promotion, tenure

## Abstract

In this editorial, I share advice and general principles based on recent experiences as a mentor and evaluator for early-career microbiology and immunology faculty seeking promotion and tenure. I outline 10 recommendations covering research, service, teaching, and mentoring. In addition, I encourage nuanced conversations with colleagues to strategically navigate the unique promotion and tenure processes at different institutions. I hope that these practical tips will assist early-career faculty in attaining promotion and tenure, contributing to long-term scientific and career advances.

## EDITORIAL

Thanks to a kind nomination by early-career faculty colleagues, I was recently awarded the 2023 International Cytokine and Interferon Society Mentorship Award. This recognition coincided with my promotion to full professor and involvement on promotion and tenure committees within my university and as an external evaluator. These experiences as mentor, candidate, and evaluator prompted me to compile advice for early-stage microbiology and immunology faculty with the hope that it will help strategize for promotion and tenure.

## RESEARCH

1. Stay focused. Being a recognized expert in a specific disease, molecule, cell type, pathogen, etc. is a common tenure requirement. Having clear expertise demonstrated by your publication record will also lead to invitations to collaborate, speak at other universities and conferences, and review grants and papers on this topic. Furthermore, external evaluators will have an easier time writing a positive letter in support of your promotion if they can identify a coherent research theme in your publications. Keeping this in mind can help you decide whether to take on new projects or collaborations pre-tenure.
2. Align your publication strategy with departmental expectations. Many departmental promotion policies list an expectation for a specific number of papers published per year in journals with a minimum impact factor threshold. In this case, I would advise mentees that the clearest path to promotion is to meet the stated metric and to submit for publication as soon as they have a significant conclusion with enough supporting figures to fill a credible manuscript. However, if you are at an institution where publishing in prestige journals is expected or where specific target journals are outlined, then you should likely wait until you can get into those preferred journals.
3. Demonstrate independence. Conventional wisdom states that one should stop publishing with a postdoctoral advisor to demonstrate independence. If your past mentors are listed on your papers, you may not get full credit from evaluators for leadership of those manuscripts regardless of the author order. If you continue work with a past mentor, make sure you have enough independent projects and papers such that you are not reliant on the collaborative works to qualify for promotion.
4. Collaborate with productive people. A rule of thumb for choosing collaborations is that if the collaborator is prolific with their publishing, then your contributions will likely materialize quickly into a publication that will aid your case for promotion. If your involvement is substantial, it may be reasonable to request a co-corresponding author designation, which can boost your total number of corresponding authorships.
5. Know the funding expectations of your institution. The specific types of grants expected by your department and institution should be your primary focus. Seek clear guidance from departmental colleagues as to what extent junior investigator grants or society grants will be valued in promotion and tenure decisions. Knowing, for example, whether a non-renewable NIH grant (e.g., DP2) is considered equivalent to an R01, or whether a society grant without overhead costs would substitute for an NIH grant, is essential information to help you prioritize grant submissions and to prevent surprises when it comes time for promotion.

## SERVICE

- Provide service to your field and university. Service at the national level via journal reviewing/editing, grant reviewing, or service on professional society committees can demonstrate your respected reputation to evaluators. Do not hesitate to nominate yourself for committees or to ask senior colleagues for help in being invited to these experiences. At the local level, committee service is likely mandatory, and service in one-time tasks, such as poster judging or pilot grant reviewing, can add to your list of service without an ongoing time commitment. While these roles are expected, they are unlikely at many institutions to tip the scale toward promotion if you are deficient in research and funding, so strategically saying no to demanding chores while performing enough service to maintain collegiality is an important balance.
- Avoid service to journals with predatory practices. It has become common for professors at all levels to be invited as editors at open-access journals with predatory practices. Thus, service to these journals is not an indicator of a strong reputation and may instead indicate naivete given the massive time commitment that these duties can entail. While I shall not name the for-profit publishers I refer to, they are characterized by excessive publication of review articles and themed issues and have exceedingly low rejection rates. In contrast, service to society journals should be sought out since surpluses from these journals support science advocacy, grants, and conferences.
- Host or meet with as many guest lecturers as possible. A great way to network in your field is to host senior experts for seminars at your institution. Additionally, your colleagues who are hosting speakers will be appreciative if they can rely on you to help fill their guests’ itineraries. These meetings are sources of advice and ideas, and these experts will be your paper/grant reviewers and conference organizers for years to come. They may also be asked to provide one of your external evaluation letters for promotion. If these opportunities are not readily available, organizing online seminars with meetings afterward has become common practice.

## TEACHING AND MENTORING

- Know your teaching expectations and do your best. The amount of classroom teaching required for promotion varies between institutions and departments. Speak with colleagues to determine what is expected in terms of classroom hours and strive to create clear and compelling lectures for your students. Even if not required, ask for student and peer evaluations and incorporate suggestions in future classes so you can demonstrate that you are constantly improving.
- Invest in trainee success. Helping trainees to publish, compete for fellowships/awards, present at conferences, and graduate should be top priorities. Discussing the unique career goals of each lab member can help you tailor a strategy to advance them to their next desired career stage. Beyond altruistic reasons for helping trainees to become better candidates for their next positions, you are judged by their accomplishments, and their successes stay on your record for the rest of your career. Attending mentorship and diversity training workshops can help you improve your mentoring capabilities and demonstrate to your students, colleagues, fellowship grant reviewers, and promotion evaluators that you are working to create a strong training environment.

While these tips are meant to be general principles, each circumstance and career is unique. I hope that this advice will spark further nuanced conversations between early-career faculty and their colleagues to better understand and navigate the path to promotion and tenure at their institutions. By sharing our insights and experiences, we can collectively improve this important aspect of academic life.

